# Associations Between Substance-Related Concerns and Psychosocial Burden Outside the SIPAT Substance-Use Domain in Lung Transplant Candidates: A Retrospective Single-Center Study

**DOI:** 10.3390/jcm15155847

**Published:** 2026-07-27

**Authors:** Aleksandra Stańska, Wojciech Karolak, Jacek Wojarski, Sławomir Żegleń

**Affiliations:** 1 Division of Quality of Life Research, Department of Psychology, Faculty of Health Sciences, Medical University of Gdańsk, 80-210 Gdańsk, Poland; 2 Department of Cardiac & Vascular Surgery, Faculty of Medicine, Medical University of Gdańsk, 80-210 Gdańsk, Poland; 3 Division of Pulmonology, Faculty of Medicine, Medical University of Gdańsk, 80-210 Gdańsk, Poland

**Keywords:** lung transplantation, psychosocial assessment, Stanford Integrated Psychosocial Assessment for Transplantation, SIPAT, substance use, alcohol, smoking, candidate evaluation

## Abstract

**Background:** Psychosocial assessment is an important component of lung transplant evaluation. Substance-related concerns may coexist with broader psychosocial vulnerabilities, but this relationship is difficult to evaluate when substance-use indicators and psychosocial scores contain overlapping information. We aimed to characterize psychosocial risk in lung transplant candidates and examine whether alcohol-, nicotine-, and illicit drug-related concerns were associated with psychosocial burden outside the SIPAT substance-use domain. **Methods:** We retrospectively analyzed 491 adult lung transplant candidates admitted for their first inpatient evaluation at the University Clinical Center in Gdańsk, Poland, between December 2021 and November 2025. All patients underwent routine psychosocial consultation, including the Stanford Integrated Psychosocial Assessment for Transplantation (SIPAT), completed using a locally developed Polish translation. To avoid conceptual and statistical overlap with substance-related indicators derived from Domain D, we calculated a non-substance psychosocial score as the sum of Domains A, B, and C. Three logistic regression models adjusted for age and sex examined associations with alcohol-, nicotine-, and illicit drug-related concerns. Holm correction was applied across the three primary tests. **Results:** The mean age was 57.2 years (SD = 10.7), and 40.5% of participants were women. Most candidates were classified as excellent or good (89.4%). Alcohol-, nicotine-, and illicit drug-related concerns were documented in 39.5%, 43.8%, and 13.4% of candidates, respectively. In complete-case models (*N* = 489), each five-point increase in the non-substance psychosocial score was associated with higher odds of alcohol-related concerns (OR = 1.26, 95% CI 1.07–1.48, *p* = 0.006; Holm-adjusted *p* = 0.017) and nicotine-related concerns (OR = 1.22, 95% CI 1.04–1.43, *p* = 0.014; Holm-adjusted *p* = 0.028). No significant association was observed for illicit drug-related concerns (OR = 0.81, 95% CI 0.62–1.02, *p* = 0.090). Male sex was associated with alcohol-related concerns (OR = 2.16, 95% CI 1.47–3.19, *p* < 0.001). **Conclusions:** Alcohol- and nicotine-related concerns were associated with broader psychosocial burden extending beyond the SIPAT substance-use domain. These findings suggest that substance-related concerns may coexist with difficulties in readiness, social support, or psychological functioning rather than representing isolated behavioral findings. Because this study was cross-sectional and used a non-validated local translation, the results should not be interpreted as evidence of psychometric or predictive validity.

## 1. Introduction

For patients with advanced pulmonary disease, lung transplantation may offer a substantial survival benefit, although long-term outcomes remain limited by medical, behavioral, and psychosocial complications. Contemporary registry data indicate that approximately 55–60% of recipients survive five years after transplantation [1]. Accordingly, International Society for Heart and Lung Transplantation recommendations call for a multidimensional candidate evaluation that includes medical, surgical, and psychosocial factors [2].

Psychosocial concerns identified before transplantation may influence treatment engagement and subsequent adaptation to a complex post-transplant regimen. Relevant factors include nonadherence, psychiatric symptoms, insufficient or unstable social support, and alcohol, nicotine, or illicit substance use [3,4,5,6,7,8]. Structured assessment may help transplant teams identify potentially modifiable vulnerabilities and determine which candidates require additional education, psychological care, addiction-focused intervention, or practical support [2,3,4,9,10,11].

The Stanford Integrated Psychosocial Assessment for Transplantation (SIPAT) provides a clinician-rated framework for organizing this information across four areas: readiness and illness management, social support, psychological stability and psychopathology, and lifestyle and substance use [12]. It produces domain scores, a total score, and an overall candidate classification. Previous studies have linked higher SIPAT scores with selected psychosocial or medical outcomes, although results differ across organs and clinical settings, and some studies have not confirmed associations with adherence or rejection [9,12,13,14,15].

However, most available SIPAT research has been conducted in mixed solid-organ cohorts, while evidence concerning lung transplant candidates remains comparatively limited [9,11,12,13,15]. Substance-related concerns are particularly relevant during transplant evaluation, but they may occur alongside broader difficulties involving treatment readiness, social support, and psychological stability. Evaluating this relationship requires separating substance-use indicators from the psychosocial score used as the explanatory variable, thereby avoiding direct overlap with SIPAT Domain D.

Against this background, the present study aimed to describe the distribution of SIPAT scores, candidate categories, and substance-related concerns in a large single-center cohort of lung transplant candidates. The primary analytical aim was to examine whether alcohol-, nicotine-, and illicit drug-related concerns were associated with psychosocial burden outside the substance-use domain, operationalized as the combined score of SIPAT Domains A, B, and C. We additionally examined differences in SIPAT scores according to age, sex, and primary pulmonary diagnosis.

## 2. Methods

### 2.1. Setting and Participants

This retrospective cross-sectional analysis used routinely collected clinical data from 491 adults undergoing their initial inpatient lung transplant evaluation at the Lung Transplantation Unit or the Clinic of Pulmonology, University Clinical Center in Gdańsk, Poland. Evaluations were conducted between December 2021 and November 2025 as part of the center’s standard transplant qualification pathway.

Inclusion in the analytic cohort required a completed psychosocial consultation and complete coding of the SIPAT item scores, domain scores, total score, candidate category, and substance-related indicators used in the present study. Age, sex, and primary pulmonary diagnosis were obtained from the medical documentation. SIPAT data were complete for all included candidates, whereas age was unavailable for two individuals. The “Missing/not specified” diagnostic category reflects absent documentation of the primary pulmonary diagnosis and not missing psychosocial data.

Study data were extracted between January and March 2026 and anonymized before analysis. No research-specific assessment or modification of clinical care was introduced. The same clinical cohort has previously been examined in relation to transplant listing status and psychosocial prehabilitation [16]. That publication focused on listing outcomes, cluster profiles, and discriminatory performance of SIPAT. The present study addresses a separate question concerning associations between clinician-documented substance-related concerns and psychosocial burden calculated without Domain D; listing outcomes, cluster analyses, and ROC analyses are not repeated here.

### 2.2. Psychosocial Assessment

Psychosocial evaluations were completed by clinical psychologists with experience in transplant medicine and advanced pulmonary disease. Information was obtained through a semi-structured interview and review of the patient’s medical documentation, supplemented, when clinically necessary, by consultation with the treating team. The psychologist subsequently completed the SIPAT rating and prepared a concise psychosocial summary for consideration by the multidisciplinary lung transplant board. These evaluations formed part of routine clinical care.

#### Stanford Integrated Psychosocial Assessment for Transplantation (SIPAT)

SIPAT is a structured clinician-rated instrument developed to organize psychosocial information relevant to transplant candidacy and clinical management [12]. Higher item scores represent greater psychosocial concern. Its items are assigned to four domains: Domain A, covering readiness for transplantation and illness management; Domain B, describing the availability and reliability of social support; Domain C, addressing psychological stability, psychiatric history, and coping; and Domain D, covering lifestyle and substance-related factors.

For each candidate, item scores were summed to obtain the four domain scores and the overall SIPAT score. Candidates were also assigned to the original global categories: Excellent, Good, Minimally acceptable, Poor, or High-risk candidate.

The assessment was completed using a locally prepared Polish translation of the original English instrument. This version has been used in routine practice at our center but has not undergone a formal process of cultural adaptation or psychometric validation. The original scoring structure was retained. Because the instrument is subject to use and intellectual-property restrictions, the locally translated wording is not reproduced in the present article.

Internal consistency was examined descriptively using Cronbach’s alpha for all scored SIPAT items and separately for Domains A–D. These coefficients were calculated to characterize the present dataset and were not treated as evidence of formal validation of the Polish translation.

### 2.3. Substance-Related Indicators

Three binary indicators were derived from the relevant SIPAT Domain D items:

- alcohol-related concern (yes/no),
- illicit drug-related concern (yes/no),
- nicotine-related concern (yes/no).

A concern was coded as present when the relevant SIPAT item received a score greater than zero, indicating that a current or past substance-related issue had been documented during routine psychosocial assessment. Accordingly, these broad indicators included different levels and timeframes of concern and did not necessarily indicate current dependence, active use, or a formal substance-use disorder. They should therefore be interpreted as clinician-documented psychosocial concerns rather than diagnostic prevalence estimates.

Because these indicators were derived from Domain D, neither Domain D nor the total SIPAT score were used as an explanatory variable in analyses of substance-related concerns.

### 2.4. Statistical Analysis

Descriptive analyses and non-parametric comparisons were conducted using IBM SPSS Statistics, version 23.0 (IBM Corp., Armonk, NY, USA). Logistic regression models, confidence intervals, and Holm-adjusted *p*-values were calculated using R, version 4.5.3 (R Foundation for Statistical Computing, Vienna, Austria).

Given the ordinal and skewed distribution of SIPAT scores, we used non-parametric tests for group comparisons:

- Spearman’s rank correlation to assess the association between age and overall SIPAT score;
- Mann–Whitney U test to compare overall SIPAT scores between women and men;
- Kruskal–Wallis tests to compare overall and domain SIPAT scores across primary lung disease categories (with inspection of mean ranks to describe patterns where omnibus tests were significant).

Alcohol-, nicotine-, and illicit drug-related indicators were summarized using counts and percentages. To examine whether substance-related concerns were associated with psychosocial burden outside the substance-use domain, a non-substance psychosocial score was calculated as the sum of SIPAT Domains A, B, and C. This score included readiness and illness management, social support, and psychological stability but excluded all Domain D items.

Three binary logistic regression models were estimated with alcohol-related concern, illicit drug-related concern, and nicotine-related concern as dependent variables. Each primary model included age, sex, and the non-substance psychosocial score. Because the interpretation of a one-point difference was relatively small, odds ratios were additionally expressed per five-point increase in the non-substance score. The three primary tests involving the non-substance psychosocial score were adjusted using the Holm method.

Regression analyses were conducted using complete cases. Two candidates had missing age data; therefore, the adjusted regression models included 489 participants. Results are presented as odds ratios (ORs), 95% confidence intervals (CIs), and *p*-values.

Domain D, the total SIPAT score, and the global SIPAT category were not entered as predictors of substance-related indicators.

All tests were two-tailed, with *p* < 0.05 considered statistically significant. Analyses involving diagnostic groups were considered exploratory.

## 3. Results

### 3.1. Sample Characteristics

The analytic cohort included 491 lung transplant candidates. Age was documented for 489 participants and averaged 57.20 years (SD = 10.73; range 19–78). The sample comprised 199 women (40.5%) and 292 men (59.5%). COPD/emphysema and idiopathic or nonspecific interstitial lung disease were the two largest diagnostic groups (Table 1). These baseline characteristics were previously reported in an analysis of transplant listing status [16] and are summarized here to provide context for the present substance-related analyses.

### 3.2. SIPAT Scores and Candidate Categories

Total SIPAT scores were complete for all 491 participants and ranged from 0 to 45 (M = 12.38, SD = 6.84). Overall, 439 candidates (89.4%) were classified as Excellent or Good, whereas 52 (10.6%) were classified as Minimally acceptable or Poor (Table 2). The distribution of total scores and global SIPAT categories has been described previously for this cohort [16] and is reported here because it defines the psychosocial context in which the substance-related associations were examined.

### 3.3. Internal Consistency

Cronbach’s alpha for the 21 scored SIPAT items was 0.693, and the standardized alpha was 0.718. Domain-level coefficients were 0.488 for Domain A, 0.615 for Domain B, 0.596 for Domain C, and 0.490 for Domain D. Thus, internal consistency was higher for the total score than for the individual domains, while the domain-level coefficients were low to moderate. These estimates are reported descriptively and should not be interpreted as constituting formal psychometric validation of the Polish translation.

### 3.4. Associations with Age, Sex and Underlying Diagnosis

Age was not significantly related to the overall SIPAT score (Spearman *rho* = −0.035, *p* = 0.440). There were also no differences in the overall SIPAT score between women and men (Mann–Whitney *U* = 28828.50, *Z* = −0.15, *p* = 0.884).

Across diagnostic categories, there was a statistically significant but modest difference in the overall SIPAT score (Kruskal–Wallis χ^2^(14) = 29.60, *p* = 0.009). Mean ranks tended to be higher in patients with COPD/emphysema, bronchiectasis and pneumoconiosis, and lower in those with cystic fibrosis and rare ILD, but the pattern was not strictly linear.

For the SIPAT domains, only the lifestyle and substance use domain showed clear differences between diagnostic groups (χ^2^(14) = 48.19, *p* < 0.001). Domain scores for readiness and illness management, social support and living environment, and psychological stability did not differ significantly across diagnoses (all *p* ≥ 0.216).

### 3.5. Substance-Related Concerns and Their Psychosocial Correlates

Alcohol-related concerns were documented in 194 candidates (39.5%), nicotine-related concerns in 215 candidates (43.8%), and illicit drug-related concerns in 66 candidates (13.4%). These indicators reflected any current or past concern recorded through a non-zero score on the corresponding SIPAT Domain D item and did not necessarily represent active use or a formal substance-use disorder.

The non-substance psychosocial score, calculated as the sum of Domains A, B, and C, ranged from 0 to 35, with a mean of 10.33 (SD = 5.77) and a median of 9.

In logistic regression models adjusted for age and sex, a higher non-substance psychosocial score was associated with alcohol-related concerns and nicotine-related concerns. Each five-point increase was associated with 26% higher odds of an alcohol-related concern (OR = 1.26, 95% CI 1.07–1.48, *p* = 0.006) and 22% higher odds of a nicotine-related concern (OR = 1.22, 95% CI 1.04–1.43, *p* = 0.014). Both associations remained statistically significant following Holm correction for the three primary models (adjusted *p* = 0.017 and *p* = 0.028, respectively).

The non-substance psychosocial score was not significantly associated with illicit drug-related concerns (OR per five-point increase = 0.81, 95% CI 0.62–1.02, *p* = 0.090). Male sex was associated with higher odds of an alcohol-related concern (OR = 2.16, 95% CI 1.47–3.19, *p* < 0.001), but not with illicit drug- or nicotine-related concerns. Age was not significantly associated with any of the three outcomes. The adjusted models included 489 complete cases (Table 3).

## 4. Discussion

In this single-center cohort of 491 lung transplant candidates, most patients were classified within the Excellent or Good SIPAT categories, while alcohol-, nicotine-, and illicit drug-related concerns were frequently documented during routine assessment. The principal finding was that alcohol- and nicotine-related concerns were associated with psychosocial burden extending beyond the SIPAT substance-use domain. After Domain D was excluded from the explanatory score, higher combined scores for readiness and illness management, social support, and psychological stability remained associated with alcohol- and nicotine-related concerns. No corresponding association was observed for illicit drug-related concerns. These results suggest that alcohol- and nicotine-related concerns may coexist with broader psychosocial vulnerabilities rather than occurring as isolated behavioral findings.

### 4.1. Contextual Distribution of Psychosocial Risk

Most candidates were classified within the Excellent or Good SIPAT ranges, and the mean total score indicated generally limited psychosocial burden. This distribution is consistent with earlier lung and mixed-organ studies in which very high global psychosocial risk was relatively uncommon [9,12,13,14,15,17]. Neither age nor sex was associated with the total SIPAT score, supporting the use of individualized assessment rather than demographic assumptions.

Total scores differed modestly across pulmonary diagnostic categories, but the clearest diagnostic variation occurred in Domain D. This pattern may partly reflect differences in previous tobacco exposure and other health-related behaviors associated with diseases such as COPD or pneumoconiosis. Domains addressing readiness, social support, and psychological functioning did not differ significantly across diagnoses. Because several disease categories were small and the diagnostic analyses were exploratory, these findings should not be interpreted as evidence that pulmonary diagnosis determines overall psychosocial functioning.

### 4.2. Substance-Related Concerns and Broader Psychosocial Vulnerability

The associations observed after excluding Domain D indicate that substance-related concerns were not explained solely by the direct contribution of substance-use items to the total SIPAT score. Alcohol- and nicotine-related concerns were associated with higher psychosocial burden across the remaining domains, even after adjustment for age and sex. Expressed per five-point increase, the corresponding increases in odds were 26% for alcohol-related concerns and 22% for nicotine-related concerns.

Clinically, this pattern suggests that substance-related concerns may accompany difficulties in treatment readiness, coping, social resources, or psychological functioning. A documented alcohol- or nicotine-related concern may therefore warrant broader psychosocial evaluation rather than being addressed solely as an isolated behavioral risk. The absence of a statistically significant association for illicit drug-related concerns may reflect the smaller number of affected candidates, greater heterogeneity of the indicator, or a genuinely different psychosocial pattern.

These findings should nevertheless be interpreted cautiously. Substance-related indicators were derived from the same routine SIPAT assessment and were not confirmed using independent measures such as AUDIT, structured diagnostic interviews, cotinine testing, or other biological verification. Excluding Domain D from the explanatory score removed direct construct overlap but did not eliminate potential shared-method variance arising from a single clinical assessment.

### 4.3. Reliability and Multidimensionality of the Instrument

The total SIPAT score showed borderline internal consistency, whereas the coefficients calculated separately for the four domains were low to moderate. These results do not establish the reliability or validity of the locally translated Polish version. Cronbach’s alpha may also be of limited interpretative value for SIPAT domains because they contain relatively few items and intentionally combine clinically distinct characteristics rather than interchangeable indicators of a single latent construct. Nevertheless, the low domain-level coefficients require caution and prevent the domains from being interpreted as psychometrically validated subscales.

Accordingly, these internal-consistency estimates should be interpreted descriptively and should not be considered evidence of psychometric validation of the Polish version. Formal evaluation of the Polish version will require standardized translation and cultural adaptation procedures, independent external measures, test–retest assessment, inter-rater reliability, and analyses of longitudinal clinical outcomes.

### 4.4. Interpretation and Clinical Context

The study was not designed to establish predictive validity or to compare SIPAT with an unstructured psychosocial interview. Its contribution lies in showing that alcohol- and nicotine-related concerns were associated with psychosocial vulnerabilities located outside the SIPAT substance-use domain. This distinction is clinically relevant because it suggests that documented substance-related concerns may identify candidates who require assessment and support across several psychosocial areas.

SIPAT results should nevertheless remain one component of a comprehensive psychosocial evaluation. Clinical interpretation should integrate the interview, medical records, collateral information, multidisciplinary discussion, and changes in the patient’s functioning over time rather than relying exclusively on a total score, domain score, or categorical threshold.

### 4.5. Limitations and Future Directions

Several limitations should be acknowledged. First, this retrospective study was based on routine data from one transplant center, which limits generalizability. The same 491-candidate cohort was used in our previous publication concerning transplant listing status and the role of SIPAT within a psychosocial prehabilitation pathway [16]. That report analyzed listing outcomes, SIPAT-based psychosocial profiles, cluster membership, and ROC performance. The current study addresses a different research question, uses substance-related concerns as dependent variables, and evaluates their associations with a psychosocial score from which Domain D was excluded. Listing-status outcomes and the principal statistical models from the previous article are not reproduced here. Although our sample is relatively large for a lung transplant cohort, the number of patients in some diagnostic subgroups remains small. Therefore, comparisons involving rare diagnostic categories (e.g., cystic fibrosis, LAM and other uncommon diagnoses) should be interpreted cautiously and considered exploratory rather than definitive. Second, we used a locally translated version of SIPAT that has not yet undergone full psychometric validation in Polish. While our translation followed the original items closely and the overall pattern of associations matches those reported in the literature, formal validation work, including test–retest reliability and factor structure, is still needed. The present study should therefore not be interpreted as a validation study, and the reported reliability coefficients cannot establish equivalence between the locally translated version and the original English instrument.

Third, the substance-related indicators were derived from SIPAT Domain D rather than from independent diagnostic instruments, self-report questionnaires, or biological verification. Although Domain D was excluded from all explanatory scores used in the primary regression analyses, the predictors and outcomes were obtained during the same clinician-rated assessment. Shared-method variance therefore cannot be excluded.

The broad binary indicators represented any non-zero score on the corresponding SIPAT Domain D item and combined different levels and timeframes of concern. They did not consistently distinguish remote history, recent use, current use, dependence, or relapse risk and should not be interpreted as formal diagnoses or prevalence estimates.

Fourth, two participants had missing age data, resulting in a complete-case regression sample of 489 candidates. Fifth, the study was cross-sectional and did not include post-transplant adherence, rejection, graft function, survival, or other longitudinal outcomes. It therefore does not provide evidence concerning the predictive validity of SIPAT. Finally, several pulmonary diagnostic groups contained small numbers of patients, and comparisons involving rare diseases should be considered exploratory.

Future research should use a formally adapted Polish SIPAT version together with independent validated measures of substance use and psychological functioning, inter-rater and test–retest reliability assessment, and prospective post-transplant outcomes.

## 5. Conclusions

In this single-center cohort, alcohol- and nicotine-related concerns were associated with broader psychosocial burden extending beyond the SIPAT substance-use domain. Higher combined scores for readiness and illness management, social support, and psychological stability were associated with alcohol- and nicotine-related concerns after adjustment for age and sex, whereas no significant association was observed for illicit drug-related concerns. These findings suggest that substance-related concerns may coexist with vulnerabilities across multiple psychosocial areas and support comprehensive rather than narrowly substance-focused assessment. Because the indicators were clinician-coded, the Polish SIPAT translation was not formally validated, and no longitudinal outcomes were examined, the findings should be interpreted as exploratory and require confirmation using independent measures and prospective designs.

## Figures and Tables

**Table 1:** Sociodemographic and clinical characteristics (*N* = 491).

Characteristic	Category/Value	*n*	%
**Age (years)**	Mean ± SD (range)	57.20 ± 10.73 (19–78)	-
**Sex**	Female	199	40.5
	Male	292	59.5
**Underlying diagnosis**	COPD/emphysema (incl. A1AT, asthma-COPD)	165	33.6
	Idiopathic/nonspecific ILD (incl. IPF)	121	24.6
	Pulmonary arterial/chronic pulmonary hypertension	40	8.1
	ILD in connective tissue disease (CTD-ILD)	36	7.3
	Hypersensitivity pneumonitis (HP)	30	6.1
	Post-COVID chronic lung disease (ILD/mixed)	21	4.3
	Sarcoidosis (± other)	17	3.5
	Other/mixed (cardiac, oncologic, etc.)	17	3.5
	Bronchiectasis (non-CF)	10	2.0
	Pneumoconiosis	8	1.6
	LAM	5	1.0
	Asthma (without clear COPD/ILD)	5	1.0
	Cystic fibrosis/CF/bronchiectasis	3	0.6
	Other rare ILD (e.g., LIP)	1	0.2
	Missing/not specified	12	2.4

***Abbreviations:*** COPD, chronic obstructive pulmonary disease; ILD, interstitial lung disease; IPF, idiopathic pulmonary fibrosis; CTD-ILD, connective tissue disease-related interstitial lung disease; HP, hypersensitivity pneumonitis; A1AT, alpha 1 antitrypsin deficiency; CF, cystic fibrosis; LAM, lymphangioleiomyomatosis; LIP, lymphocytic interstitial pneumonia; COVID, coronavirus disease 2019. ***Note:*** Baseline characteristics of this cohort were previously reported in relation to transplant listing status and psychosocial prehabilitation [16] and are presented here to contextualize the current analyses. Age data were missing for two participants; descriptive age statistics are based on 489 candidates.

**Table 2:** Overall SIPAT scores and candidate categories.

SIPAT Measure	Value
Overall SIPAT score–mean ± SD (range)	12.38 ± 6.84 (0–45)
SIPAT category–Excellent candidate	90 (18.3%)
SIPAT category–Good candidate	349 (71.1%)
SIPAT category–Minimally acceptable candidate	48 (9.8%)
SIPAT category–Poor candidate	4 (0.8%)

***Note:*** The distribution of SIPAT total scores and candidate categories in this cohort was previously reported in relation to transplant listing status [16] and is included here for contextual purposes.

**Table 3:** Associations between non-substance psychosocial burden and substance-related concerns.

Outcome	Predictor	OR	95% CI	*p*-Value	Holm-Adjusted *p* *
**Alcohol-related concern**	Age, per year	1.01	0.99–1.02	0.488	—
	Male sex	2.16	1.47–3.19	<0.001	—
	Non-substance psychosocial score, per 5 points	1.26	1.07–1.48	0.006	0.017
**Illicit drug-related concern**	Age, per year	1.00	0.98–1.02	0.921	—
	Male sex	1.30	0.76–2.27	0.344	—
	Non-substance psychosocial score, per 5 points	0.81	0.62–1.02	0.090	0.090
**Nicotine-related concern**	Age, per year	1.00	0.98–1.02	0.987	—
	Male sex	1.36	0.94–1.97	0.103	—
	Non-substance psychosocial score, per 5 points	1.22	1.04–1.43	0.014	0.028

***Abbreviations:*** CI, confidence interval; OR, odds ratio. Female sex was the reference category. The non-substance psychosocial score was calculated as the sum of SIPAT Domains A, B, and C. Models were adjusted for age and sex and included 489 complete cases. * Holm-adjusted *p*-values apply only to the three primary tests of the non-substance psychosocial score.

## Data Availability

The clinical dataset cannot be deposited in a public repository because of patient-confidentiality and institutional data-protection requirements. Access to appropriately de-identified data may be considered following a reasonable request to the corresponding author, institutional authorization, and execution of a suitable data-sharing agreement.

## Abbreviations

A1AT	alpha 1 antitrypsin deficiency
CF	cystic fibrosis
COPD	chronic obstructive pulmonary disease
COVID	coronavirus disease 2019
CTD-ILD	connective tissue disease-related interstitial lung disease
HP	hypersensitivity pneumonitis
ILD	interstitial lung disease
IPF	idiopathic pulmonary fibrosis
ISHLT	International Society for Heart and Lung Transplantation
LAM	lymphangioleiomyomatosis
LIP	lymphocytic interstitial pneumonia
OR	odds ratio
SD	standard deviation
SIPAT	Stanford Integrated Psychosocial Assessment for Transplantation
SPSS	Statistical Package for the Social Sciences
